# Randomized comparison of HARVesting the Left Internal Thoracic Artery in a skeletonized versus pedicled technique: the HARVITA trial—study protocol

**DOI:** 10.1093/icvts/ivae045

**Published:** 2024-03-21

**Authors:** Hannes Abfalterer, Elfriede Ruttmann-Ulmer, Michael Grimm, Gudrun Feuchtner, Sarah Maier, Hanno Ulmer, Sigrid Sandner, Daniel Zimpfer, Torsten Doenst, Martin Czerny, Matthias Thielmann, Andreas Böning, Mario Gaudino, Matthias Siepe, Nikolaos Bonaros

**Affiliations:** Department of Cardiac Surgery, Medical University of Innsbruck, Innsbruck, Austria; Department of Cardiac Surgery, Medical University of Innsbruck, Innsbruck, Austria; Department of Cardiac Surgery, Medical University of Innsbruck, Innsbruck, Austria; Department of Radiology, Medical University of Innsbruck, Innsbruck, Austria; Institute of Medical Statistics and Informatics, Medical University of Innsbruck, Innsbruck, Austria; Institute of Medical Statistics and Informatics, Medical University of Innsbruck, Innsbruck, Austria; Department of Cardiac Surgery, Medical University of Vienna, Vienna, Austria; Department of Surgery, Division of Cardiac Surgery, Medical University of Graz, Graz, Austria; Department of Cardiac Surgery, University of Jena, Jena, Germany; Department of Cardiovascular Surgery, University of Freiburg, Freiburg, Germany; Department of Thoracic and Cardiovascular Surgery, West-German Heart & Vascular Center, University Hospital Essen, University of Duisburg-Essen, Essen, Germany; Department of Cardiovascular Surgery, University Hospital Giessen, Giessen, Germany; Department of Cardiothoracic Surgery, Weill Cornell Medicine, New York City, NY, USA; Department of Cardiac Surgery, University Hospital Bern, University of Bern, Switzerland; Department of Cardiac Surgery, Medical University of Innsbruck, Innsbruck, Austria

**Keywords:** Left internal thoracic artery, Skeletonized versus pedicled, Harvesting technique, Graft patency rate

## Abstract

**Figure:**
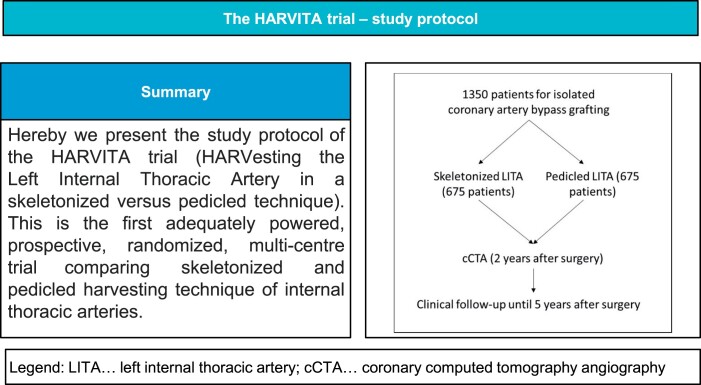

Latest research has indicated a potential adverse effect on graft patency rates and clinical outcomes with skeletonizing the left internal thoracic artery. We aim to provide a prospective, randomized, multicentre trial to compare skeletonized versus pedicled harvesting technique of left internal thoracic artery concerning graft patency rates and patient survival. A total of 1350 patients will be randomized to either skeletonized or pedicled harvesting technique and undergo surgical revascularization. Follow-up will be performed at 30 days, 1 year, 2 years and 5 years after surgery. The primary outcome will be death or left internal thoracic artery graft occlusion in coronary computed tomography angiography or invasive angiography within 2 years (+/- 3 months) after surgery. The secondary outcome will be major adverse cardiac events (composite outcome of all-cause death, myocardial infarction and repeated revascularization) within 1 year, 2 years and 5 years after surgery. The primary end point will be compared in the modified intention-to-treat population between the two treatment groups using Kaplan–Meier graphs, together with log-rank testing. Hereby, we present the study protocol of the first adequately powered prospective, randomized, multicentre trial which compares skeletonized and pedicled harvesting technique of left internal thoracic artery regarding graft patency rates and patient survival.

## OBJECTIVES

Since the landmark study by Loop *et al.* in 1986 [1], the left internal thoracic artery (LITA) is the preferred bypass conduit to the left anterior descending artery (LAD), owing to its survival benefit over the saphenous vein graft (SVG), which has also been noted in other observational studies [2, 3] and in 1 small randomized trial [4].

Since occlusion of the proximal LAD more often leads to fatal myocardial infarction than occlusion of non-LAD coronary vessels (except from the left main coronary artery), the LAD has an extremely important role in myocardial revascularization [1].

Therefore, current European and American guidelines [5, 6] recommend the use of the LITA to the LAD to improve patient outcomes.

Two techniques exist for surgical harvesting of the LITA during coronary artery bypass grafting (CABG): pedicled and skeletonized harvesting techniques [7]. While a pedicle contains the artery, together with its accompanying veins, fatty tissue and endothoracic fascia, in skeletonized harvesting technique, only the artery is harvested.

Skeletonizing the internal thoracic artery (ITA) may be more time-consuming and more challenging, but it provides a longer graft and better free flow [8]. Furthermore, various studies have described the reduced incidence of sternal wound infections with the skeletonized harvesting technique [9–13]. On the other hand, when only one ITA is used, skeletonization does not provide any additional effect on preventing sternal wound complications [9]. Deep sternal wound infections in particular are associated with increased mortality and morbidity [14], but they are caused by multiple factors, not solely by the ITA harvesting technique [15]. Lazar *et al.* was able to eliminate any form of sternal wound infection by 3 perioperative measures: perioperative intravenous antibiotics, topical vancomycin applied to the sternal edges and tight glycaemic control [16].

Besides the potential beneficial effects, skeletonizing the ITAs is thought to be more prone to injury [17]. Latest research by Lamy *et al.* and Gaudino *et al.* has noted a potential adverse effect of skeletonizing the LITA on graft patency rates and clinical outcomes [18, 19]. In a post hoc analysis of the COMPASS trial, Lamy *et al.* saw a significantly reduced short-term graft patency at 1 year and a significantly higher risk for major adverse cardiac events (MACE) at 23 months after CABG for skeletonized harvesting technique. In a post hoc analysis of the ART trial, Gaudino *et al.* did not provide data on graft patency rates, but at 10 years, the risk for MACE was significantly higher for skeletonized versus pedicled ITA grafts. A difference in the 10-year mortality rate was not seen. Interestingly, the impaired outcome was only observed with surgeons who had enrolled fewer than 51 patients in the study, implying that the surgeon’s experience plays a key role. Furthermore, a significant learning curve has been described for LITA graft harvesting [20].

To date, no adequately powered randomized trial has been performed to investigate the influence of the harvesting technique of ITA on graft patency rates and clinical outcomes. Due to the ongoing debate on the potential adverse outcome of skeletonized harvesting technique of ITA, only prospective randomized trials will show whether skeletonizing of ITA is a safe procedure or contains potential adverse effects.

Therefore, with the HARVITA trial, our goal is to provide a prospective, randomized, multicentre trial to compare patency rates following skeletonized versus pedicled harvesting technique of LITA.

## METHODS

### Study design

The HARVITA trial is a 2-arm, prospective, randomized, multicentre clinical trial, the goal of which is to evaluate the impact of the LITA harvesting technique on patency rates. All patients who are referred for isolated CABG will be screened for inclusion and exclusion criteria. Informed consent will be required for eligible patients. Patients will be randomized to skeletonized or pedicled harvesting technique of LITA. Coronary computed tomography angiography (cCTA) will be performed to evaluate LITA graft status 2 years (+/- 3 months) after surgery. Follow-up will be performed at 30 days, 1 year, 2 years and 5 years after surgery.

### Primary hypothesis

The primary hypothesis is as follows: The harvesting technique of LITA (skeletonized versus pedicled) is associated with a difference in the rate of death or LITA graft occlusion within 2 years (+/- 3 months) after surgery.

## ELIGIBILITY

### Inclusion criteria

- Primary isolated CABG patients with multivessel disease (defined as ≥ 70% stenosis of the LAD and ≥ 50% stenosis of the circumflex and the right coronary territory, with or without  ≥ 50% stenosis of the left main artery)

### Exclusion criteria

- Age > 80 years
- Planned CABG without using the LITA
- Preoperative mediastinal radiation therapy
- Emergency operation
- Minimally invasive coronary artery bypass surgery
- Any concomitant cardiac or non-cardiac procedures
- Previous cardiac surgery
- Known contrast agent allergy
- Severe stenosis of the left subclavian artery/left-sided subclavian steal syndrome
- Chronic kidney disease (glomerular filtration rate < 60 ml/min/1.73m^2^)
- Life expectancy of less than 5 years
- Pregnancy
- Hyperthyroidism
- Iodine allergy

### Intraoperative exclusion criteria

- Y/T graft off the LITA graft
- LITA sequential grafting
- LITA target vessel other than LAD

### Randomization, stratification and enrolment

All patients having a planned isolated CABG procedure are screened according to the inclusion and exclusion criteria. Those patients who are eligible and have given informed consent will be randomized to 1 of the 2 treatment arms (skeletonized or pedicled harvesting technique).

Patients will be randomized in a 1:1 fashion. Permuted block randomization with variable block sizes and stratification by centre will be performed with a web-based randomization system in order to achieve an equal distribution in both groups. Log will be held for all screened patients with reasons for inclusion and exclusion.

### Surgical procedure

Surgery should take place within 4 weeks of randomization. It will be carried out via a median sternotomy and either on-pump or off-pump. Harvesting of the LITA is performed by surgeons who are technically capable of both harvesting techniques and who have harvested at least 50 ITAs. The LITA is harvested with electrocautery or with a harmonic scalpel, independently of allocated harvesting techniques and according to the established method in each centre. Only topical, but not intravasal, application (in order to decrease the risk of a potential endothelial damage) of spasmolytic agents will be used. If the patient is randomized to the skeletonized harvesting technique, only the LITA itself will be harvested. If the pedicled harvesting technique is used, the LITA, together with its accompanying veins and parts of the endothoracic fascia, will be harvested, creating a 1- to 2- cm broad pedicle. Through an incision in the pericardium, the LITA is brought intrapericardially and then anastomosed with running suture to the LAD. In case of pedicled harvesting technique, the pedicle is stabilized without tension at the height of the anastomosis with sutures at the surface of the heart, to avoid twisting of the pedicle. Any target vessel for the LITA other than the LAD is against protocol. For the LITA, a sequential or T/Y graft configuration is not allowed. The LITA is primarily used as an in situ graft. The remaining diseased coronary vessels (≥1.5 mm and target vessel stenosis ≥50%) will receive SVG, radial artery (RA) or right ITA (RITA). The SVG can be harvested using either the open (conventional or no-touch) or the endoscopic technique. The RA can be harvested in open or endoscopic technique; in both cases as a pedicle. The RITA can be harvested using either the skeletonized or the pedicled technique, independent of the randomization process. Surgeons are encouraged to attach the proximal part of the SVG and/or the RA to the ascending aorta. It is recommended not to use the RA that has been used for coronary angiography (CAG) prior to the operation. It is also recommended to anastomose the RA to a target vessel with high-grade stenosis. After de-cannulation and administration of protamine, transit time flow measurements are used for final evaluation of all grafts. All transit time flow measurements are performed at a mean arterial pressure of 70 to 80 mmHg, as far distally as safely possible. The mean graft flow (ml/min), pulsatility index and mean arterial blood pressure (mmHg) are recorded.

### Recommendations to prevent sternal wound infections

Sternal wound infections are associated with high mortality and morbidity [14]. The European Association for Cardio-Thoracic Surgery and the American Association for Thoracic Surgery provide guidelines for the prevention and treatment of sternal wound infections [14, 21]. We recommend applying the following measures to both treatment groups (skeletonized and pedicled harvesting technique) in order to prevent the occurrence of sternal wound infections:

- Routine screening for nasal carriers of *Staphylococcus aureus;*
- Applicaton of topical mupirocin to the nares in all patients without negative screening for staphylococcus using a nasal swab within 24 h of the operation and for up to 5 days postoperatively;
- Administer continuous insulin therapy to keep the blood glucose level < 180 mg/dl within the first 24 h after surgery or for the duration of the stay in the intensive care unit;
- Administer a cephalosporin (either cefuroxime or cefazolin) as a first choice 60 min prior to the skin incision and for up to a maximum of 72 h (individual institutional protocols are accepted);
- Use a topical application of vancomycin to the bone edges immediately after a median sternotomy and prior to sternal closure;
- Avoid the use of bone wax.

### Postoperative treatment

Postoperative treatment will be carried out according to local standards and current guidelines [22]. Treatment with antiplatelet agents should be restarted within 24 h after the operation, in case there is no concern regarding surgical bleeding. If a RA was used as a graft, the decision to use spasmolytic medication (agent, duration and time of initiation) will be left to local practice. Secondary prophylaxis should be carried out according to current guidelines [5, 23, 24]. We generally recommend the use of aspirin as an antiplatelet agent (indefinitely), the use of angiotensin-converting-enzyme inhibitors/angiotensin receptor blockers (sartane), the use of a beta blocker and the use of a statin for guideline-conforming secondary prophylaxis. In case of dual antiplatelet therapy (off-pump, previous acute coronary syndrome, previous elective/acute coronary stent implant), we recommend the use of aspirin in combination with clopidogrel to ensure uniformity.

## OUTCOME MEASURES

### Primary outcome

Death or LITA graft occlusion in cCTA or invasive angiography within 2 years (+/- 3 months) after surgery.

### Secondary outcomes

MACE-free survival (composite outcome of all-cause death, myocardial infarction and repeated revascularization) within 1 year, 2 years and 5 years after surgery.

Additional secondary outcomes include the following:

- Death/LITA graft occlusion (in cCTA or invasive angiography)/intraoperative LITA graft injury within 2 years (+/- 3 months) postoperatively;
- LITA graft occlusion (and LITA graft dysfunction) in cCTA or invasive angiography at 2 years (+/- 3 months);
- LITA graft occlusion at cCTA or invasive angiography for patients with cCTA or invasive angiography for clinical reasons;
- Repeated revascularization at 2 years and 5 years after surgery;
- Repeated revascularization of the left anterior descending artery (LITA target vessel revascularization) at 2 years and 5 years after surgery;
- Sternal wound complications at 1 year after surgery;
- Composite end point of the LITA graft occlusion/dysfunction (cCTA or invasive angiography), myocardial infarction and repeat revascularization within 2 years (+/- 3 months) after surgery;
- Perioperative outcome at 30 days.

### Further analyses

- Primary and secondary end points for male versus female sex;
- Primary and secondary end points according to the severity of target vessel stenosis (moderate 50 - <70%, severe ≥70% or occlusion);
- Competing risk analyses.

### Follow-up

Patients will receive cCTA examinations at 2 years (+/- 3 months) postoperatively. One week prior to the cCTA scan, blood samples (glomerular filtration rate, creatinine and thyroid stimulating hormone for the upcoming cCTA; LDL-cholesterol and HbA1c for follow-up) will be collected. At 30 days, 1 year, 2 years and 5 years postoperatively, phone calls will be used for follow-up (Table 1). cCTA should be performed within a time span of 6 months (-3 months to + 3 months) for 2 year’s cCTA. If cCTA or invasive angiography is performed for clinical reasons (e.g. signs of acute or chronic ischaemia, acute myocardial infarction, heart failure or recurrence of symptoms) prior to the above-mentioned time interval (> 3 months prior to the cCTA at 2 years) and the LITA graft is not occluded, cCTA will be performed according to protocol. If cCTA or invasive angiography is performed for other reasons prior to the above-mentioned time interval (> 3 months prior to cCTA at 2 years) and the LITA graft is occluded, cCTA will not be performed, and the findings of the cCTA/CAG will be used for statistical analysis. If CAG is performed for other reasons within the above-mentioned time intervals (≤ 3 months prior to the cCTA at 2 years) and the LITA graft does or does not show LITA graft occlusion, the cCTA will not be repeated, and the findings of the CAG will be used for statistical analysis. In the case of occlusion or dysfunction of other grafts rather than the LITA, patients with clinical symptoms and/or pathological findings noted from non-invasive testing should be referred for invasive angiography. This decision will be left to the clinical assessment of the participating centres.

**Table 1:** The follow-up process

Follow-up	30 days	1 year	2 years	5 years
Telephone interview	x	x	x (2 years + 3 months)	x
cCTA			x (2 years +/− 3 months)	

cCTA: coronary computed tomography angiography.

### Coronary computed tomography angiography

At each participating centre, two independent experienced radiologists, blinded to patient data (especially the allocated harvesting cohort) [but not to the type of graft (LITA/RA/RITA/SVG) and their target vessels], will evaluate the cCTA results according to graft patency status. Graft status will be analysed for all bypass grafts. Graft patency by cCTA will be determined and classified as 1 = patent, 2 = dysfunctional and 3 = 100% occlusion.

In case of equal assessment of graft status by the 2 independent radiologists, no further assessment is necessary. In case of unequal assessment, cCTA image data will be sent anonymized as a DICOM file to the core centre. The cCTA will be assessed by a third experienced radiologist [blinded to patient data (especially allocated harvesting cohort) but not to the type of graft (LITA/RA/RITA/SVG) and their target vessels] at the core centre. This is considered as the final evaluation.

In cases with inconsistent results and if asked by the core centre, invasive angiography will be performed.

Cardiac computed tomography angiography will be performed in each centre using a CT scanner with ≥ 64 slices. At the Medical University Innsbruck, a 128-slice dual-source CT (Definition FLASH or DRIVE, Siemens Healthineers, Erlangen, Germany) with a detector collimation of 2 × 64 × 0.6 mm and a rotation time of 0.28 s will be used, together with high-pitch (3.2) scanning (Flash mode). Scans will be triggered into arterial phase using bolus tracking (threshold of 100 HU, ascending aorta) and by injecting an intravenous iodine contrast agent [iopromide (Ultravist 370), Bayer Healthcare, Berlin, Germany, 70–120 ml, depending on the patient’s body mass index]. Prospective electrocardiography triggering will be applied, and images will be reconstructed at an end-diastolic phase (70% of the RR-interval). Thinslice images will be reconstructed with a 0.75-mm slice width (increment, 0.4) and transferred to three-dimensional postprocessing software (SyngoVIA, Siemens Healthineers, Erlangen, Germany) for cCTA image analysis. Estimated radiation exposure will be 1–3 mSv.

Beta blockers may be given to lower the patient’s heart rate, pending on the centre’s individual internal guidelines (scanner-specific), prior to the scan. Patients will be advised not to drink coffee prior to the CT examination (in order to avoid an increase in heart rate).

### Outcome definitions

- LITA graft occlusion in cCTA: absence of contrast detection in the lumen of the graft, indicating a 100% occlusion of LITA graft in cCTA;
- LITA graft dysfunction in cCTA: suspicion of LITA graft dysfunction in cCTA, either anatomical [anatomical stenosis ≥ 50% (for example, due to plaques, stricture) at anastomotic site or in the course of the graft], functional (due to competitive flow) or unclear (diffuse small-sized vessel without clear anatomical obstruction);
- LITA graft occlusion in CAG: complete occlusion (100%) of the LITA graft;
- LITA graft dysfunction in CAG: ≥ 50% stenosis of the LITA graft, string sign of the graft due to competitive flow or graft spasm;
- Intraoperative LITA graft injury: surgeon’s decision not to use LITA as a conduit after the harvesting process;
- MACE: composite outcome of all-cause death, myocardial infarction and repeated revascularization;
- All-cause death: death from any cause (cardiac or non-cardiac) from the time of the surgical procedure;
- Cardiac death: death due to myocardial infarction, cardiogenic shock, sudden cardiac death or cardiac arrhythmias;
- Non-cardiac death: death from any cause other than cardiac (e.g. cancer, trauma, pulmonary embolism);
- Myocardial infarction: composition of periprocedural myocardial infarctions and non-periprocedural myocardial infarctions;
- Periprocedural myocardial infarction during CABG: defined as type 5 myocardial infarction according to the criteria of the 4th universal definition of myocardial infarction [25];
- Spontaneous myocardial infarction: defined as types 1–3 myocardial infarctions according to criteria of the 4^th^ universal definition of myocardial infarction [25];
- Repeat revascularization: any form of repeat revascularization [CABG, percutaneous coronary intervention (balloon angioplasty or stent implantation)] after the index operation;
- Target vessel repeat revascularization: any form of repeat revascularization [CABG, percutaneous coronary intervention (balloon angioplasty or stent implantation)] to the LAD after the index operation;
- Sternal wound complication: superficial or deep sternal wound infection requiring external vacuum therapy, surgical treatment including wound debridement, open vacuum-assisted therapy or sternal reconstruction with concomitant antibiotic therapy;
- Perioperative mortality: death within 30 days after the primary surgery.

### Supportive clinical centres

The following centres will participate in the trial: (i) Department of Cardiac Surgery, Medical University of Innsbruck, Innsbruck, Austria (H. Abfalterer/N. Bonaros) (core clinical centre); (ii) Department of Cardiac Surgery, Division of Surgery, Medical University of Vienna, Vienna, Austria (S. Sandner); (iii) Department of Surgery, Division of Cardiac Surgery, Medical University of Graz, Graz, Austria (D. Zimpfer); (iv) Department of Cardiac Surgery, University of Jena, Jena, Germany (T. Doenst); (v) Department of Cardiovascular Surgery, University of Freiburg, Freiburg, Germany (M. Czerny); (vi) Department of Thoracic and Cardiovascular Surgery, West-German Heart & Vascular Center, University Hospital Essen, University of Duisburg-Essen, Essen, Germany (M. Thielmann); (vii) Department of Cardiovascular Surgery, University Hospital Giessen, Giessen, Germany (A. Böning); (viii) Department of Cardiac Surgery, University Hospital Bern, Bern, Switzerland (M. Siepe).

## STATISTICS

### Study design and objectives

This is a 2-arm, prospective, randomized, observer-blinded, multicentre clinical trial, designed to evaluate the impact of harvesting technique of LITA on graft occlusion-free survival. The primary end point is defined as death or LITA graft occlusion identified via cCTA or invasive angiography within 2 years (+/− 3 months). The secondary end points include MACE- (composite outcome of all-cause death, myocardial infarction and repeated revascularization) free survival, occlusion rate and other graft-related outcomes. LITA graft occlusion-free survival and MACE-free survival are treated as time-to-event variables with observation time ranging from date of surgery (time zero) to either date of event or censoring date.

### Sample size rationale/number of patients

A sample size estimation was performed using data of a post-hoc analysis of the COMPASS trial [18]. In this trial at 1 year, LITA-to-LAD graft occlusion occurred in 7.3% (21/289) of skeletonized and in 3.4% (25/725) of pedicled grafts (the COMPASS trial did not provide 2-year results). In addition, within 2 years, 5 of 1014 patients died. Rounding up this numbers, we consider event rates of 4% (pedicled) versus 8% (skeletonized) at 2 years as a realistic, conservative scenario for our study. In order to detect this difference of 4% (corresponding to a hazard ratio of 0.49), as statistically significant with a two-sample log-rank test, a sample size of 558 patients in each treatment group is needed, assuming a type I error of 0.05 (alpha = 5%) and a power of 0.8 (beta = 20%), requiring 62 events (death or LITA occlusion) in total. To account for dropouts and withdrawals, we increased the sample size to 675 patients in each group, resulting in a total sample size of 1350 patients for the trial.

### Study population

The following populations will be used for statistical analysis: (i) The intention-to-treat population, which comprises all individuals who are randomized to 1 of the arms of the HARVITA trial, regardless of adherence, treatment or protocol deviations; (ii) modified intention-to-treat population, which includes individuals who are randomized, undergo the surgical procedure and have a LITA graft anastomosed to the LAD.

## DATA ANALYSIS

### Demographic and baseline characteristics

A flow chart will be produced, showing the number of patients screened, excluded, randomized, receiving surgery and having follow-up. Baseline demographic data will be presented as absolute numbers with percentages for categorical variables and as mean+/- standard deviation or median (interquartile range) for continuous variables.

### Efficacy analysis

The primary end point of LITA graft occlusion-free survival will be compared between the 2 treatment groups using Kaplan–Meier graphs and a centre-stratified two-sample log-rank test. In addition, Cox proportional hazards regression analysis adjusting for clinically relevant confounders, will be performed. Hazard ratios and their 95% confidence intervals will be estimated.

The primary efficacy hypotheses will thus be formulated as:

H_0_: hazard ratio_skeletonized vs pedicled_ = 1

H_1_: hazard ratio_skeletonized vs pedicled_ ≠ 1

The primary efficacy analysis will be performed in the modified intention-to-treat population population.

MACE-free survival and other secondary end points that follow the time-to-event format will be analysed with Kaplan–Meier test,log-rank test and Cox proportional hazards regression analysis. Categorical end points will be compared between treatment groups using the χ^2^ test. *P*-values < 0.05 will be considered statistically significant; however, formal significance testing will be applied to the primary hypothesis only. Statistical tests for secondary end points will be applied in a descriptive manner only.

### Safety analysis

Safety variables will be summarized using descriptive statistics and tabulated by treatment group.

A safety monitoring committee comprised of 3 independent consultants (2 consultants in cardiac surgery, 1 consultant in cardiology) will meet annually and inspect the follow-up data. The primary safety outcome composed of death, myocardial infarction and stroke, as well as the secondary safety outcome, composed of periprocedural major complications (reoperation due to bleeding, perioperative myocardial infarction, dialysis, tracheostomy, stroke and deep sternal wound infections) will be compared. In case of a greater than 10% difference between the 2 treatment groups, the safety monitoring committee, together with the trial steering committee, will temporarily pause the randomization of further patients until a final decision is made. This final decision could be either the early termination of the trial, a change in the study protocol or the continuation of the trial.

### Software

All statistical analyses will be performed with SPSS Version 28 (IBM Corporation, Armonk, NY, USA), MedCalc Version 19.4, GraphPad Prism version 9.0. and R 3.2.2 (The R Foundation for Scientific Computing, Vienna, Austria).

### Ethics

Permission for this study was obtained from the local institutional review board on 1 December 2023 (Medical University of Innsbruck) (EK Nr: 1135/2023). All participating centres will apply for approval of the study protocol from their local institutional review board before proceeding with enrolling patients in the trial.

### Registration

The HARVITA trial is registered at ClinicalTrials.gov (NCT05931783).

## Data Availability

The data underlying this study will be available upon request from the corresponding author.

## ABBREVIATIONS

CABG: Coronary artery bypass grafting
CAG: Coronary angiography
cCTA: Coronary computed tomography angiography
CT: Computed tomography
ITA: Internal thoracic artery
LAD: Left anterior descending artery
LITA: Left internal thoracic artery
MACE: Major adverse cardiac events
RA: Radial artery
RITA: Right internal thoracic artery
SVG: Saphenous vein graft
